# Psychological consequences for family caregivers of patients receiving hemodialysis: threat or opportunity?

**DOI:** 10.1186/s40359-021-00667-7

**Published:** 2021-10-07

**Authors:** Abbas Ebadi, Seyedeh Azam Sajadi, Seyed Tayeb Moradian, Roghayeh Akbari

**Affiliations:** 1 Behavioral Sciences Research Center, Lifestyle Institute, Nursing Faculty, Baqiyatallah University of Medical Sciences, Tehran, Iran; 2 Department of Nursing Management, Faculty of Nursing, Aja University of Medical Sciences, Kaj St., Shariati St, Tehran, Iran; 3 Atherosclerosis Research Center, Nursing Faculty, Baqiyatallah University of Medical Sciences, Tehran, Iran; 4 Department of Nephrology, School of Medicine, Health Research Institute, Babol University of Medical Sciences, Babol, Iran

**Keywords:** Dialysis, Care, Family caregiver, Psychological effects

## Abstract

**Background:**

Family caregivers of patients receiving hemodialysis experience physical and psychological disorders. They are unfortunately neglected. The aim of this study was to explain the psychological consequences for family caregivers of patients receiving hemodialysis.

**Methods:**

This qualitative inductive conventional content analysis research approach was conducted in Tehran, Iran. Nineteen hemodialysis patient caregivers were enrolled via purposive sampling. Data collection was conducted through in-depth and semi-structured interviews until reaching data saturation. All interviews were recorded, transcribed, imported into the Open Code Software, and analyzed using the Graneheim and Lundman methods.

**Results:**

The results included two main categories: (1) threats to the psychological integrity; (2) development of capabilities. The first main category comprised the sub-categories of “care-related negative feelings and emotions,” care-related stress and its behavioral impacts on care, “psychological disorders arising from care provision,” and “impaired quality and quantity of sleep.” The second main category comprised of the sub-categories of “care-related positive feelings and emotions” and “coping strategies.”

**Conclusions:**

The present study showed that though caring for hemodialysis patients threatens the caregiver’s psychological integrity, it provides the opportunity of development capabilities.

## Background

Care is an essential issue in general health [1], and caregivers as vital and national healthcare resources [2], are at risk of contracting various diseases. Unfortunately, these care partners are neglected [3, 4]. Informal caregivers, although unpaid, are most involved in patient care, adaptation, and management of chronic diseases [5, 6]. The patients with chronic diseases are mostly taken care at home by family members [7]. Chronic kidney disease is a global health problem that entails a burden to patients and their family members [8]. Family is the best resource for patients receiving hemodialysis [9]. Family members play a fundamental role in disease management and improving the quality of life of patients with chronic kidney failure undergoing hemodialysis treatment [2, 9]. Given the healthy and intact family structure in Asian countries, families of patients treated with hemodialysis assume the roles of caregivers [2]. Iranians also profoundly adhere to traditions, and strong emotional bonds between family members are directly influenced by the family collection and taking on an extraordinary commitment to each other. Such a traditional structure is one of the critical sources of support for a patient [10]. Hemodialysis is also defined as a family illness, and family caregivers are considered partners in this therapeutic process. Family caregivers prefer the needs of patients over their own and ultimately spend less time on health-promoting behaviors that adversely affect their health status and life routines [4, 11, 12]. Likewise, the burden of dialysis patient care does not only affect the caregiver but also the patient [13] and leads to inadequate care of patients and patient abandonment [8, 14].

Although the related literature was focused on the negative aspects of care provision (stress, depression, financial loss, and low health status and quality of life), a few studies have cited positive aspects of care. These aspects include a sense of satisfaction, improved personality growth, post-traumatic growth, spiritual development, a sense of efficiency and fulfillment that give meaning to the care experience and result in lower levels of depression and stress [15–18]. There are very few studies that address both the positive and negative aspects of hemodialysis patient care.

Nurses have a crucial role in the improvement of the health care system [19]. Hemodialysis patients have special conditions compared to other chronic patients. These special conditions, including dependence on the dialysis machine, lead to changes in the lives of their family caregivers. Understanding the experiences of family members by nurses can contribute to providing better family-centered care as one of the primary goals of holistic healthcare and opens a window for interventional research to improve the psychological status of family caregivers. Considering that qualitative research studies are helpful in the analysis of experiences gained by individuals, this study was conducted to explain the consequences for family caregivers of patients receiving hemodialysis.

## Methods

This qualitative inductive conventional content analysis research approach was conducted in Tehran, Iran. Qualitative research is based on the paradigm of naturalism. In the naturalistic approach, the researcher is the research instrument. Naturalistic researchers spend so much time interacting directly with participants to observe, describe and interpret their experiences [20].

Among the qualitative studies, qualitative content analysis with insight into complex interactions is one of the best methods for analyzing qualitative data in various fields [21]. The inductive conventional content analysis is used when research theories or findings are absent or limited. This method can provide direct data resulting from study participants imposing no predetermined beliefs, categories, or theories [22]. The study setting was the room specified for a family member in the dialysis ward, hospital campus, and the office of the Kidney Foundation, which was chosen based on the participant's willingness. The participants were recruited based on the purposive sampling technique. The researcher interviewed 19 key informants of 15 family caregivers and four others (including one patient receiving hemodialysis, one nurse, one physician, and one head of the Kidney Foundation) who were in close contact with family caregivers and knew their problems.

The study participants spoke Persian aged over 18 years as hemodialysis patients’ family members with 6-month experience in patient care, highest involvement in the care of patients, endowment with rich experience, ability to talk about the research questions, and have the willingness to participate and cooperate in the study and recount their experiences. The exclusion criteria in this study were the unwillingness to participate further in this research project. No one refused or withdrew after giving their agreement.

The data were collected using face-to-face deep semi-structured interviews with open-ended questions by the corresponding author (Ph.D. degree in Nursing and expert in Qualitative Research) in a comfortable location for the participants and mostly in a private room. The researcher with no previous relationship with family members, introduced herself to the participant and explained the purpose of the research. After gaining trust and asking warm-up questions, the following guide questions were asked from family caregivers. For example: “How do you feel as you take care of the patient?”, “What were the psychological impacts *related to* hemodialysis *patient care* on you?”*. *“*How was your situation before from the time you did not take care of the patient?*”

- *Other participants were also asked *“*What are the psychological effects of taking care hemodialysis patients as a family caregiver?*”

The participants were asked to provide more examples or clarify their reasons of this topic. In addition, probing questions along with open-ended ones were raised to understand more details of the participants’ experiences. For instance, “*What do you mean by that?*” At the beginning of each interview, the researcher explained the purpose of the study to the participants. The participants were assured regarding the confidentiality of data and voice recordings. The mean duration of the whole interview was 45 min.

The interviews were continued until data saturation had been reached, meaning no further data and new categories were obtained. All the interviews were recorded, transcribed verbatim, and imported into Open Code Software [23]. Finally, the data were analyzed based on the steps set forth by Graneheim and Lundman by two researchers (first and corresponding authors with experience in qualitative data processing) as follows;

1. Transcribing the whole interview immediately after its administration
2. Reading the entire transcript of the interview to reach an overview of its content
3. Determining the units of meaning and initial codes
4. Grouping the same initial codes into more comprehensive categories
5. Specifying the latent content of the data and extracting the themes [24].

The interviews were transcribed and reread several times to immerse in the data and obtain a general sense of the interview transcripts. Then, the codes were extracted by specifying the particular parts of the transcripts encompassing the fundamental concepts. After that, the researcher entered into the course of categorization through determining and writing down initial perceptions and analyses and simultaneously specified the titles of the codes directly from the transcripts during the progression of the analysis process. Next, the codes were grouped into various categories based on their similarities and differences, and tencoding was performed to explain the themes containing the components of the psychological dimension.

The researchers conducted the manifest and latent content analysis. Constant comparison analysis was utilized to review the extracted sub-categories and categories. The researchers inductively and independently created codes, sub-categories, and main categories. Ambiguities were controlled by checking the transcripts with the participants immediately after the interview. Besides, field notes were taken during the data collection process.

One characteristic of qualitative studies is trustworthiness. Lincoln and Guba proposed four criteria to increase the study's trustworthiness [25]. To enhance the credibility; the researchers considered good communication skills with participants, allocation of sufficient time for data collection, immersion and used data (field notes, interviews with family caregivers and medical staff) and researcher triangulation (involvement of all authors in reviewing the codes, subcategories and, categories).

To increase transferability, a maximum variation regarding the two dimensions of various social and demographic characteristics of caregivers (age, gender, marital status, level of education, employment status, income status, insurance status, type of care, living with patient, disease infection, number of diseases, and number of months for patient care) and a variety of social and demographic characteristics of patients (age, gender, marital status, level of education, employment status, income status, insurance status, and number of diseases) was observed much.

To observe the confirmability, the findings were approved by some participants (member check) and two qualitative method researchers (peer check). The dependability was accommodated via maintaining documentation related to the study, by recording interviews, using direct quotes, and disclosure the codes.

This research received approval from the Ethics Committee of the Baqiyatallah University of Medical Sciences, Tehran, Iran, with the code: IR. BMSU.REC.1395.38. The researchers considered the Declaration of Helsinki and the Committee on Publication Ethics (COPE). For instance, informed written and oral consent and interview recordings were obtained from all study participants. They were voluntary and were ensured of the data's confidentiality and their right to withdraw from the study.

## Results

Nineteen participants were included in this study. The mean (range) age and care provision of family caregivers were 42.16 ± 48 (27–78 years) and 46.33 ± 97.24 (6–84 months), respectively. The majority of participants were females (60%), married (66.7%), housewife and employee (60%), literate (86%), with inadequate income (53.3%), history of the disease (60%), and lived in the same room with the patient (86%). Six were the patient’s wife, 7 were offsprings and 2 were the patient’s parents. The characteristics of the patients cared for by these family caregivers were as follows. The mean (range) age of care recipients was 60.37 ± 16 (32–90 years). The majority were females (56.3%), married (81.3%), housewife and employee (62%), literate (80%), with inadequate income (62.3%) and, having a history of another disease (62%).

The mean (range) age, Total Work Experiences and Experiences of treatment and care of hemodialysis patients of other informants were 49 (48–62 years), 24 (23–30 years), and 13 (12–14) years, respectively. They were all married, and two were men.

The study findings included six sub-categories, two main categories, and one theme depicted in Table 1.

**Table 1:** Subcategories and main categories

Theme	Main categories	Sub-categories
Psychological tides	Threats to the psychological integrity	Care-related negative feelings and emotions
		Care-related stress and its behavioral impacts on care
		Psychological disorders arising from care provision
		Impaired quality and quantity of sleep
	Development of capabilities	Care-related positive feelings and emotions
		Coping strategies

### Threats to the psychological integrity

The first category obtained from the data in this study was “threats to the psychological integrity,” comprised the sub-categories of “care-related negative feelings and emotions,” care-related stress and its behavioral impacts on care, “psychological disorders arising from care provision,” and “impaired quality and quantity of sleep.”

#### Care-related negative feelings and emotions

Care of the patient with a chronic disease by family members imposes psychological pressures due to emotional attachments. This can lead to the emergence of negative feelings and emotions regarding care provision. Compassion fatigue, the shadow of silence at home, a sense of helplessness, being in trouble, sense of captivity in the hospital, frequent referrals to hemodialysis department, being locked in care, regretfulness, depression, distress from patient’s sufferings, neglect of caregivers by others, being affected by pressures of care, and a sense of boredom with caring were among the negative feelings and emotions about care provision expressed by the study participants. One stated that:

- *Whenever you see your mother, someone you love, someone who helped you all these long years, and someone now doing so, is suffering from all these pains; you are also affected by her pains and droped off… there is no pain like the one that your mother is suffering, but you can do nothing to help her* (P 10)*.*
- *The patient receiving hemodialysis and whose daughters were taking care of her said: My daughter wants to go to the market and says something. She says we do not have a mother. We have to do all our work ourselves. You all say I'm sick. They are suffering from my illness* (P 15)*.*

#### Care-related stress and its behavioral impacts on care

Care has all the characteristics of chronic stress because it is endowed with high levels of unpredictability and uncontrollability. These factors also help to create secondary stress in different aspects of life including work and family relationships. In this respect, the study participants acknowledged that patients receiving their care did not have stable and predictable clinical conditions. In addition, uncooperative patients in terms of no compliance with diet and medication regimen, failed fistula, hospital-acquired infections, inadequate hemodialysis, concerns about kidney transplant, problems with providing medications, worries about patient’s increasing critical conditions, need for continuous patient monitoring, and requirements for staying with the patient could impose severe stress on caregivers. These stresses come with confusion, worry, anxiety, restlessness, fear, fatigue, shock, and extreme panic of losing patients. One stated that:

- *Hemodialysis is worse than cancer because the hemodialysis patient can suddenly go into critical conditions or coma and suffer from high blood pressure. You get nervous because the situations of such patients are not predictable* (P 11)*.*
- *The dialysis nurse who has been working in the dialysis ward for several years says: Even in the ward, even though we are all there and everything is ready in terms of medicine and nursing procedures, suddenly, the patient gets sick, the pressure drops, he becomes lethargic. I have seen that patient's daughters, are very shocked and feel bad, and are under a lot of stress. They think their patient died under the dialysis machine. They are very upset. Sometimes we even had to take them out of the ward, because that tension disturbs the peace inside the hospital ward* (P 17).

#### Psychological disorders arising from care provision

Family caregivers go through numerous psychological disorders. *Informants mentioned* stress, depression, sadness, anxiety, irritability, impatience, aggression, mental fatigue, mental confusion, and loss of concentration. One stated that:

- *I used to be a teacher, and I was very contented. I used to be lively and energetic, but these days I do not have the same feelings. My children always tell me why I am so. My sister tells me you used to be tolerant and relaxed. Everybody knew me as a quiet person, full of peace. However, now, I have changed my mood, I am irritable, I suddenly shout, I wonder myself, I feel sad, but I cannot control it. I think they are all due to the pressures I am bearing* (P 7).

#### Impaired quality and quantity of sleep

Sleep quality and quantity disorder accompany concerns arising from patients' needs for care following critical conditions at night, patient monitoring, and reduced hours of sleep to compensate for performing multiple roles. The study participants also highlighted disorders such as the inability to sleep, consumption of sleeping pills, nightmares, inadequate sleep, Dreams, frequent sleep arousals, and dissatisfaction with quality of sleep. One stated that:

- *He tells me day and night: I am suffering from shortness of breath. We have a personal oxygen device at home. He feels better during the day, but he goes into dangerous conditions at night. He continually asks me to help him stand up and move. He has interrupted my sleep.* (P 12).

### Development of capabilities

The second main category extracted from this study was the “development of capabilities”, comprised the sub-categories of “care-related positive feelings and emotions” and “coping strategies.”

#### Care-related positive feelings and emotions

Different people have various subjective assessments of care. Despite being stressful and complicated, some caregivers have a positive evaluation in this respect. A sense of resoluteness, release from sorrows, peacefulness, feeling of joy and happiness, love for care, and enjoying care were among the care-related positive feelings and emotions outlined by the study participants. One stated that:

- *I love to take care of my mother. I do not think there is something more enjoyable than taking care of a mother. As soon as I sit next to her, I gain lots of energy* (P 4).

#### Coping strategies

Any change in human life requires adaptation and the use of coping strategies to overcome problems. Such coping strategies are sometimes passive strategies and sometimes appear inactive ways in caregivers. Family caregivers in this study mentioned passive and active coping strategies. Passive coping strategies include belief in fate and destiny and compatibility with passing time and routines. Active coping strategies include playing sports, practicing music, religious coping (reciting the Holy Qur’an, attendance in religious ceremonies, prayers, and pilgrimage), increased morale, planning for routines, acceptance of the disease in the family member. And they point to familiarity with the hemodialysis machine, communication with caregivers having the same or worse conditions, comparing conditions with those of other caregivers, and adopting an unconcerned view towards life. Two participants stated that:

- *I go to the gym. I need to be in high spirits to relay it to someone else. Exercises can help me to get rid of pressures. For example, when I am angry or mentally tired, I lift weights, feel better and forget everything. Thank God because I can be happy doing exercises* (P 9).
- *Well, this is also the right way of treatment and a kind of lifestyle. You must come here to feel okay and get back. We have gotten on well with this issue. It was hard at first, but then we accepted that is not so hard, and that is not the end of the world* (P 2).

## Discussion

The results showed that positive feelings and emotions accompanied the care of patients treated with hemodialysis in some people or negative ones among other individuals. The results of some studies show that caregivers can have positive or negative perceptions about their care provision [15, 17, 26–28]. These positive and negative aspects can be related to caregivers' and care recipient's characteristics. Some factors associated with the caregivers included age, gender, type of communications, and social, economic, and cultural status and some factors related to care recipients were clinical and functional conditions [1, 26].

Care also contains all the characteristics of experiencing chronic stress. This activity comes with long-term physical and mental pressures and high levels of unpredictability and uncontrollability. These factors lead to secondary stress in different aspects of life including work and family relationships. Moreover, the stressors encountered by caregivers can cause psychological stress and impair health behaviors which stimulate physiological responses, diseases, and even death [1]. Family caregivers reported problems and disorders such as stress problems and disorders like stress, depression, anxiety, and lack of self-confidence, fatigue, social isolation, financial and communicative constraints, and reduced quality of life.

These problems have a significant effect on physical, social, and emotional well-being [13, 29–31].

The results recommended that family caregivers can adopt passive or active coping strategies in the face of care-related pressures. In this respect, the findings of a study conducted on caregivers of hemodialysis patients shed light on the factors relieving these problems, such as belief in God and attendance in religious ceremonies [27]. The study on the relationship between religious coping with depression and care burnout also demonstrated that using positive coping strategies was correlated with reduced depression and burnout care [32]. In another study, the adoption of effective coping strategies came with a decline in the burden of care among family caregivers of patients treated with hemodialysis [3]. The results of a qualitative study indicated that most strategies adopted by families of patients included avoidance strategies (caregivers’ preference to escape from the situations), search for social supports (dialogues with individuals and professionals and getting information), and problem-solving strategies (developing a strong sense of responsibility compared with the premorbid conditions [33]. Also, the findings of another study on caregivers of patients receiving hemodialysis suggested that the majority of the strategies used by this group were directly correlated with social and demographic variables [26]. In the present study, the participants also listed a variety of coping strategies.

One of the other frequently reported problems by caregivers in the present study was sleeping disorders. In this respect, the participants pointed out several disorders such as the inability to sleep, consumption of sleeping pills, nightmares and bad dreams, inadequate sleep, frequent sleep arousals, and dissatisfaction with quality of sleep. The findings of this study follow the results of other studies, and they also reported some decrease in the quantity and quality of sleep [4, 34–37].

Three themes entitled fluctuations in sleep quality based on patient’s conditions, need for vigilance to care for patients, and concerns of late-night ruminations about current and future events were extracted from a qualitative study on sleep quality among patients caregivers affected with dementia [37]. In our study, participants also shed light on the intellectual concerns of care, use of hours of sleep to compensate for performing multiple roles, and need for patient night monitoring as causes of sleep disorders.

With a view of the mentioned cases, it seems facing new and complex conditions of care can bring about ups and downs in the psychological state of caregivers, and such turbulence can be weak or strong depending on coping strategies. Therefore, the main theme of this study was psychological tides.

The strength of this study was that the researcher interviewed different people to answer the research question. These include family caregivers, the nurse, patient, and physician who interacted with the family caregivers.

This study had a limitation. The participants were likely not able to express some real-life experiences due to personal reasons. The researcher tried to control this limitation by establishing communication and gaining trust.

## Conclusion

The present study showed that though caring for hemodialysis patients threatens the psychological integrity of the caregiver, it provides the opportunity to development capabilities. Thus, psychological counseling with an emphasis on active coping strategies and improvement of the relationships between professional caregivers such as the nurses and family caregivers should be considered imperative. Because understanding the experiences of family members by nurses and other health care providers also help them provide better family-centered care as one of the primary goals of holistic care.

The mental health status of the caregiver affects the quality of patient care. Therefore, designing valid and reliable questionnaires to assess the mental health status of caregivers is essential.

## Data Availability

The datasets used and analyzed during the present study are available from the corresponding author on reasonable request.
